# Cryptococcal Pneumonia in an Immunocompetent Patient: A Rare Occurrence

**DOI:** 10.7759/cureus.29841

**Published:** 2022-10-02

**Authors:** Muhammad S Haider, Madiha Master, Arun Mahtani, Eduardo Guzzo, Ambreen Khalil

**Affiliations:** 1 Internal Medicine, Richmond University Medical Center, New York, USA; 2 Medical School, Philadelphia College of Osteopathic Medicine, Philadelphia, USA

**Keywords:** adenocarcinoma, lung cancer, fungal, immunocompetent, pneumonia, cryptococcal neoformans

## Abstract

Cryptococcosis is an invasive yeast infection commonly found among immunosuppressed patients. Pulmonary cryptococcal infection can have variable presentations ranging from pulmonary nodules and masses to consolidation. A patient can present with shortness of breath, cough, sputum production, chest pain, fatigue, and weight loss. Diagnosis can be made using fungal culture, histology, radiographic findings, and cryptococcal antigen in serum as well as in the cerebrospinal fluid. Treatment is usually with a combination or a single antifungal agent. Few cases have been reported in immunocompetent individuals.

Here we present a case of 69-year-old immunocompetent individual, who was initially seen in the outpatient clinic for dyspnea, cough, and fatigue and was treated for pneumonia. The patient remained symptomatic despite multiple courses of oral antibiotics. He was then sent for inpatient admission. CT scan was obtained that showed patchy infiltrates and consolidations, followed by bronchoscopy. The cytology confirmed adenocarcinoma. The fungal smear and culture grew *Cryptococcus neoformans*. The patient was treated with fluconazole with improvement of his symptoms before starting chemotherapy.

We are reporting this case as clinicians usually focus on bacterial etiologies in outpatient setting. Our patient, who was immunocompetent, had a new diagnosis of cryptococcal pneumonia and was also found to have lung adenocarcinoma. This case highlights the rare occurrence of this type of pneumonia in immunocompetent patients and the importance of considering fungal causes of pneumonia in patients.

## Introduction

Cryptococcal species are invasive fungi that can cause pulmonary, meningeal, or disseminated infection. The two most common species are *Cryptococcus neoformans* and *Cryptococcus gatii* [1]. Around one million cases are reported each year resulting in 625,000 deaths [2]. In the United States, around 0.4-1.3 cases per 100,000 population and 2-7 cases per 100,000 people affected with AIDS have been recorded [3]. Cryptococci are commonly found in soil that is contaminated with bird droppings or decaying wood [4,5]. Cryptococcosis usually occurs in immunocompromised individuals; however, there have been several small-scale studies or case reports describing infection in immunocompetent hosts [6,7]. The presence of polysaccharides such as glucuronoxylomannan and glucuronoxylomannogalactan in its capsule is the main virulence factor that leads to an infection [8]. Initial infection occurs due to inhalation of spores found in the contaminated soil, following which there can be hematogenous spread to different organs, especially in immunocompromised patients [9]. Reactivation of the organism once a patient becomes immunocompromised is another manner of developing cryptococcosis [10]. However, in immunocompetent individuals, cryptococcus usually remains asymptomatic due to the development of a robust immune response against the organism. Through this case report, we would like to highlight symptomatic cryptococcal infection in an immunocompetent individual and discuss in depth about symptomatology and management options.

## Case presentation

**Figure 1:**
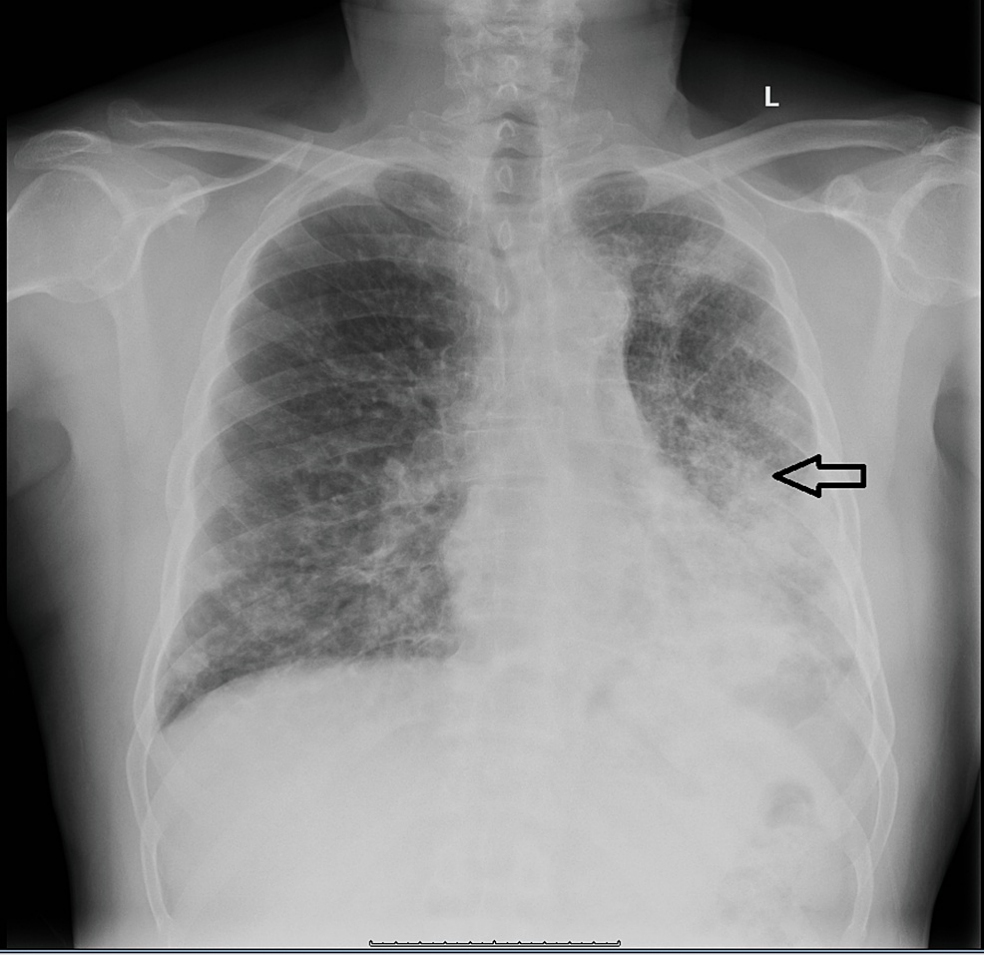
Chest x-ray posteroanterior view shows extensive patchy-to-confluent left-sided infiltrates. These are most severe in the left mid to lower lung fields with also significant patchy density in the left upper lobe. There are reticulonodular opacities involving right lower lung zone.

A 69-year-old African American male was admitted to the hospital due to outpatient treatment failure of pneumonia. The patient complained of shortness of breath on exertion, cough productive of white phlegm, and fatigue for three months. He had a past medical history of coronary artery disease, status post myocardial infarction and stent placement, hypertension, hyperlipidemia, gout, benign prostatic hyperplasia, and no signs or diagnoses that would suggest immunosuppression. The patient was a former smoker and denied any other substance use. Outpatient chest x-ray showed extensive patchy-to-confluent infiltrates in the entire left lung, but most severe in the left mid to lower regions. Infiltrates in the right lung were lesser, yet showed a diffuse coarse reticular pattern (Figure 1).

The primary care doctor diagnosed the patient with community-acquired pneumonia and prescribed amoxicillin-clavulanate and azithromycin. However, despite the full course of antibiotics, the symptoms worsened, the the infiltrates on chest x-ray persisted, and his primary care doctor advised inpatient management. 

On admission, the patient had SpO_2_ of 97% at rest in room air, which fell to 86% with ambulation, respiratory rate of 19 breaths per minute, blood pressure of 130/79 mmHg, heart rate of 105 bpm, and temperature of 98.4ºF. The examination revealed decreased breathing sounds and crackles bilaterally, with no other significant findings. The labs showed a white blood cell count of 7.9 k/UL, troponin I <0.015 ng/mL, and NT-Pro-BNP 58 pg/mL. After consultation with an infectious disease specialist, the patient was started on intravenous levofloxacin, meropenem, and vancomycin to cover for gram-negative and methicillin-resistant *Staphylococcus aureus* as well as atypical bacterial etiological agents.

**Figure 2:**
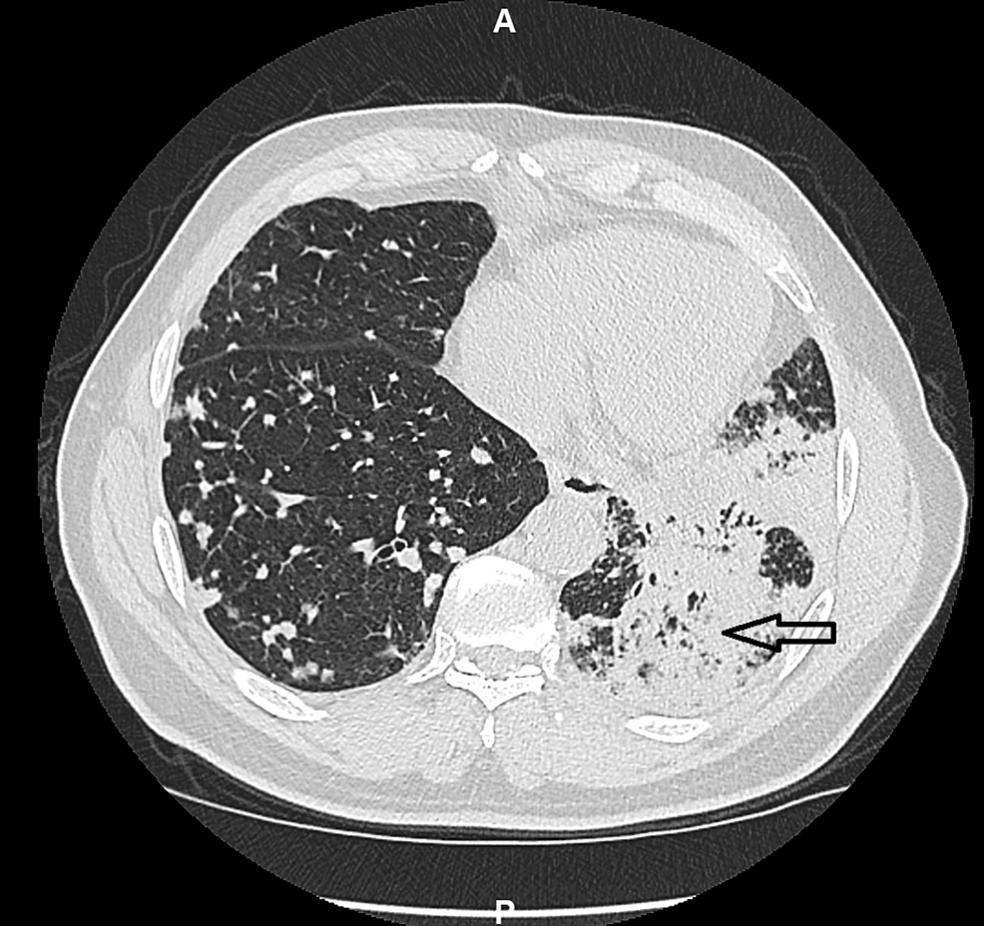
Axial section of CT chest shows extensive pneumonic infiltrates in the left lower lobe.

Further workup with blood cultures, urine culture, urine *Legionella* antigen, Quantiferon gold test for tuberculosis, serum HIV 1 and 2 antigen and antibody, and serum *Aspergillus* antigen returned negative. CT scan of the chest showed extensive consolidations in the left upper and lower lobes and innumerable nodules of 6 mm or less in size, scattered throughout both lungs, suggestive of malignancy (Figure 2). 

The patient underwent bronchoscopy, bronchial brushing, and lavage. Cytology came back positive for adenocarcinoma of the lung with positive immunostains TTF-1 and CK-7. A positron emission tomography (PET) scan was negative for metastatic disease. He remained hemodynamically stable, and we discharged him with antibiotics, supplemental oxygen, and follow-up with the infectious disease and oncology clinics. 

In the outpatient setting, the fungal smear and culture from the bronchial brushing came back positive for *Cryptococcus neoformans*, for which he received six weeks of oral fluconazole. After treatment, the patient did not need supplemental oxygen any longer, and infectious disease cleared him to start treatment for the adenocarcinoma of the lung. 

## Discussion

Cryptococcal species are encapsulated yeast fungi. They are widely found in contaminated soil, decomposing wood, pigeon, or other bird droppings [4,5]. It has become an opportunistic fungus in individuals with compromised immune systems, whereas in immunocompetent individuals, it rarely causes a clinically relevant infection [11].

The two most common species of cryptococci causing infection in humans are* Cryptococcus neoformans *and *Cryptococcus gattii.* In recent years, there have been few case reports and case series of cryptococcal pneumonia in an immunocompetent host [12,13]. However, cryptococcal pneumonia coexisting with lung adenocarcinoma is very rare. Huang J et al. reported a case series of eight patients with concomitant lung adenocarcinoma and cryptococcal pneumonia [14]. There have been other cases of similar presentation reported in the literature [15,16]. This may be due to the immune system of patients with adenocarcinoma not functioning the same way as that of a patient without cancer [17].

Diagnosing cryptococcosis can be very challenging in an immunocompetent patient. These patients often present with atypical and mild clinical symptoms. Cryptococcus species primarily manifest in the pulmonary tract and central nervous system but can also invade the skin, bones, and other parts of the body. Respiratory symptoms associated with cryptococcal infection include chest pain, dyspnea, and cough. Neurological symptoms such as stiffness of the neck and headache may also be experienced by some patients. In our case, the patient presented with respiratory symptoms including shortness of breath and productive cough for three months. Furthermore, if the infection is left untreated, cryptococcosis can result in systemic dissemination and respiratory failure, which can be life-threatening.

The status of the patient's immune system also plays an important role in the radiologic presentation of cryptococcosis in the pulmonary CT or x-ray. In immunocompromised patients, the imaging findings are nonspecific and often show a small-to-large intrapulmonary mass with a reticulonodular pattern or lung consolidation [18]. In contrast, imaging findings of an immunocompromised patient show diffuse lung consolidation due to a more severe infection [9]. Cryptococcus infection most frequently occurs in the lower lobe of the lungs [19]. Additionally, lung biopsy and tissue culture are a more definitive diagnostic tool for cryptococcosis [18].

The site and severity of the cryptococcal infection play an important role in determining the appropriate treatment. Before 1996, cryptococcal infection was treated with amphotericin B injections with or without flucytosine. However, this regimen was associated with severe side effects; therefore, the current treatment for this disease was switched to oral fluconazole which has fewer side effects but similar efficiency as the previous regimen [20]. For meningitis, a short induction course of amphotericin and flucytosine is still widely prescribed but for pulmonary infections, oral fluconazole is sufficient. Our patient was also treated with fluconazole after which he felt better and was able to wean off oxygen.

## Conclusions

Fungal pneumonia is relatively common in immunocompromised individuals. This infection can lead to fatal outcomes in these patients. Cryptococcal pneumonia is also mostly seen in immunodeficient patients. This case highlights the rare presentation of cryptococcal pneumonia in an immunocompetent individual. The case is also rare as cryptococcal pneumonia is occurring in a patient with lung adenocarcinoma. Since there have been few reports of cryptococcal pneumonia in immunocompetent hosts, especially those with lung adenocarcinoma, clinicians should always keep this disease in their differentials when encountering hard-to-treat pneumonia infections.
